# Effect of ectopic high expression of transcription factor OCT4 on the “stemness” characteristics of human bone marrow-derived mesenchymal stromal cells

**DOI:** 10.1186/s13287-019-1263-4

**Published:** 2019-06-03

**Authors:** Xiaoping Guo, Yongmin Tang, Ping Zhang, Sisi Li, Yuanyuan Chen, Baiqin Qian, Hongqiang Shen, Ning Zhao

**Affiliations:** 1 Division of Hematology-Oncology, Children’s Hospital of Zhejiang University School of Medicine, #57 Zhuganxiang Road, Yan-an Street, Hangzhou, 310003 People’s Republic of China; 2 Division of Hematology-Oncology, Zhejiang Key Laboratory for Neonatal Diseases, Children’s Hospital of Zhejiang University School of Medicine, #57 Zhuganxiang Road, Yan-an Street, Hangzhou, 310003 People’s Republic of China

**Keywords:** Transcription factor, OCT4, Overexpression, Bone marrow-derived MSCs, Stemness

## Abstract

**Objective:**

To investigate the effect of ectopic high expression of OCT4 on the stemness characteristics of bone marrow-derived mesenchymal stromal cells (BM-MSCs).

**Methods:**

BM-MSCs were collected from three de novo acute lymphoblastic leukemia (ALL) and three aplastic anemia patients (AA), which were cultivated by the whole bone marrow adherent method. Surface markers of BM-MSCs were analyzed by flow cytometry (FCM); meanwhile, growth characteristics were observed with a phase contrast microscope, and population doubling time (PDT) was calculated. The optimal generation cells (P4) were used for the subsequent experiments. Recombinant plasmid pcDNA3.1-OCT4 was constructed and transferred into ALL MSCs by liposome transfection. The cells with stable and high expression of OCT4 were selected by G418 resistance screening and subcloning, of which the expression of OCT4 was verified by FCM, cellular immunofluorescence assay (CIFA), and RT-PCR. The expression of stemness-related transcription factors (TFs) (NANOG, SOX2) and the embryonic stem cell (ESC)-related surface markers (SSEA4, TRA-1-60, and TRA-1-81) were analyzed by FCM, RT-PCR, and CIFA. Embryonic body (EB) formation was performed with the above cells, and triembryonic differentiation marker genes were evaluated by RT-PCR.

**Results:**

The primary passage of AA MSCs grew more slowly and had longer PDT (16 days on average) than ALL MSCs (10 days on average). AA MSCs presented the same typical morphology and similar expression levels of specific mesenchymal markers as ALL MSCs, whereas the latter had a much better proliferative capacity in P4 cells (*P* < 0.05). Besides, the expression levels of surface markers in ALL MSCs were slightly higher than that in AA MSCs in P4, P7, and P10 cells (*P* < 0.05). Cell lines with stable and high expression of OCT4 were successfully established from ALL MSCs, which were confirmed by CIFA, FCM, and RT-PCR. Compared with untransfected parental MSCs, the mean expression levels of TFs in OCT4 overexpression MSCs were increased from 0.63 ± 0.37% to 39.39 ± 1.85% (NANOG) and from 14.34 ± 2.44% to 91.45 ± 4.56% (SOX2). The average expression levels of ESC surface markers were increased from 3.33 ± 2.35%, 1.59 ± 1.29%, and 1.46 ± 0.86% to 84.98 ± 9.2%, 57.28 ± 6.72%, and 75.88 ± 7.35% respectively for SSEA-4, TRA-1-60, and TRA-1-81, which were confirmed by CIFA analysis. Moreover, the OCT4 overexpression MSCs could form EBs ex vivo and express ectoderm (TUBB3, WNT1), mesoderm (Brachyury, TBX20), and endoderm (SPARC) genes.

**Conclusion:**

Ectopic high expression of transcription factor OCT4 in BM-MSCs may drive them to grow as ESC-like cells with “stemness” characteristics. Single OCT4 transfection can upregulate the expression of other stemness-related transcription factors such as NANOG and SOX2.

## Background

Mesenchymal stromal cells derived from bone marrow (BM-MSCs) represent a source of pluripotent cells with high plasticity and self-renewal ability. They are non-hematopoietic multipotent stem cells with multi-lineage differentiation potential, which could differentiate into mesodermal lineages including osteogenic, chondrogenic, adipogenic, and so on [1]. BM-MSCs have already been used in various phases of clinical application and in tissue engineering. Furthermore, BM-MSCs can support hematopoiesis, are able to modulate hematopoietic microenvironment, and have been applied for the prevention and treatment of graft-versus-host disease (GVHD) [2, 3].

However, it has been reported that different culture conditions can play an important role in the biological characteristics of MSCs including gene expression, self-renewal, and multi-directional differentiation ability [4]. There are differences in the proliferative capacity and proliferation doubling time (PDT) in various tissue-specific MSCs, which will affect the hematopoiesis supporting and immunosuppressive function eventually [5, 6]. However, there are still controversies on whether MSCs have ESC-like properties or not. Compared with BM-MSCs, MSCs derived from cord blood (CB-MSCs) or amnion have greater in vitro proliferative capacity and express ESC surface markers (OCT4 and SSEA-4) [4, 6]. Thus, the biological characteristics of disease-specific BM-MSCs (for instance, those derived from ALL and AA patients) need an in-depth investigation, and the limited proliferation capability needs to be resolved for the future usage in elucidating the pathogenesis of hematology disease.

The restricted ex vivo proliferation capability of BM-MSCs and progressive loss of stemness properties during expansion hampered the putative applications albeit it is a promising cell source. The pluripotent cell-specific gene OCT4 is encoded by Pou5f1, which belongs to the family of Pou domain transcription factors, and OCT4 plays an important role in maintaining the undifferentiated state and self-renewal of ESCs [7]. It is also regarded as an indispensable and irreplaceable transcription factor in somatic cell reprogramming among the four Yamanaka factors [8]. It has been demonstrated that OCT4 expresses at very low levels in the early passage MSCs and disappears at the late passage, which indicates that OCT4 may participate in the regulation of biological behavior of BM-MSCs despite the underlying mechanism remains unclear [9]. However, whether or not the ectopic expression of OCT4 will enhance the proliferation capability or affect the stemness of BM-MSCs remains to be determined.

In this study, we tried to compare the proliferation potential of BM-MSCs derived from different types of diseases and to explore the effect of ectopic high expression of OCT4 on the stemness properties of BM-MSCs.

## Materials and methods

### Artificial synthesis of OCT4 gene sequence

The mRNA sequence of OCT4 (gene ID:5460) was obtained from GenBank, and the coding sequence of OCT4 open reading frame (ORF) was artificially synthesized and cloned into TA Vector pUC 57 (PUC57(Amp+)-OCT4) by Sangon Biotech (Shanghai) Co., Ltd. for a subsequent experiment.

### Recombinant plasmid pcDNA3.1-OCT4 construction and identification

The PUC57 (Amp+)-OCT4 vector and the eukaryotic expression plasmid pcDNA3.1 were digested with Nhe I and EcoR I (Promega), and the digested products of OCT4 and pcDNA3.1 were ligated together with T4 DNA ligase (TaKaRa, Baoshengwu, Dalian) as described by the manufacturer. Then, the ligation product was transformed into the competent *Escherichia coli* DH5α to amplify recombinant plasmid, and the correct insertion was confirmed by restriction enzyme digestion and sequencing. The purified PCR products were sequenced at least three times by Invitrogen Sequencing Department (Shanghai), and the DNA sequences were analyzed using DNAMAN software and NCBI BLAST. The accurate recombinant plasmid was named as pcDNA3.1-OCT4 and extracted according to PureLink HiPure Plasmid DNA Purification Kits (Invitrogen) instructions.

### The culture and growth evaluation of bone marrow-derived mesenchymal stromal cells

MSCs were isolated from extra bone marrow specimens of six different patients (three de novo acute lymphoblastic leukemia patients and three aplastic anemia patients) at the time of diagnosis in the Leukemia Laboratory of our hospital. The informed consents were obtained from the parents or guardians, and the ethics approval was required according to the guidelines from the Children’s Hospital of Zhejiang University School of Medicine Committee on the Use of Human Subjects in Research (approval number: 2013-GJ-003; approval date: February 25, 2013). BM-MSCs were prepared as described previously [10]. Briefly, 2–3 ml bone marrow specimens were mixed with an equal amount of DMEM (Invitrogen, Carlsbad, CA) supplemented with 10% of fetal bovine serum (FBS). The mixture was centrifuged at 1000 rpm for 5 min to discard the floating fat mass, seeded in culture flasks, and maintained at 37 °C in a 100% of humidified atmosphere. BM-MSCs were cultured in DMEM supplemented with 20% FBS, 100 U/ml of penicillin, and 100 μg/ml of streptomycin, and the medium was replaced every 2–3 days to wash away the remaining hematopoietic cells until achieving 80% of confluence. The cells were detached with 0.25% trypsin-EDTA containing 0.2% of EDTA (Haotian, Hangzhou, China). Cells were passaged at a 1:4 dilution under the same culture condition. The cell viabilities were assessed by 0.4% Trypan blue staining, the growth curves were recorded, the population doubling time (PDT) was calculated, and the growth characteristics were observed with a phase contrast microscope after each passage. The optimal generation cells (P4) were used for the subsequent experiment.

OCT4 overexpression MSCs were maintained in DMEM supplemented with 2 mM of l-glutamine (Invitrogen), 0.1 mM of β-mercaptoethanol, 1× of non-essential amino acids (NEAA; Invitrogen, Carlsbad, CA), 20% of knockout serum replacement (KSR, Invitrogen, Carlsbad, CA), 100 U/ml of penicillin, 100 μg/ml of streptomycin, and 10 ng/ml of recombinant human basic fibroblast growth factor (bFGF, Invitrogen, Carlsbad, CA) for a week, and thereafter, the cells were cultured with ES medium containing DMEM/F12 and the same supplements in 0.1% of gelatin-coated 6-well culture plate until EB formation. The cells were harvested, and the expression levels of the triembryonic layer representative marker genes were analyzed by RT-PCR.

### RT-PCR

Total RNA was isolated using Trizol® Plus RNA Purification Kit (Invitrogen, Carlsbad, USA). The integrity of the total RNA was confirmed by GeneQuant II ultraviolet illuminator spectrophotometer (Pharmacia Biotech) and 1% of agarose gel electrophoresis analysis. cDNA was synthesized from 2 μg of total RNA by M-MLV Reverse Transcriptase (Invitrogen, Carlsbad, USA) with random hexamers (Sangon, Shanghai, China) in 20 μl volume system. Platinum® Pfx DNA Polymerase (Invitrogen, Carlsbad, USA) possessing proofreading with higher fidelity was used. Amplification was performed in 50 μl volume, and PCR reaction conditions were set as follows: 35 cycles of 94 °C for 30 s denature, 62 °C (OCT4-1143bp, NANOG-1022bp) or 60 °C (GAPDH, SOX2, NANOG, OCT4-247bp, Brachyury, TUBB3, WNT1, SPARC, and TBX20) for 50 s annealing, and 72 °C for 90 s (OCT4-1143bp, NANOG-1022bp) or 30 s (the above nine genes other than OCT4-1143bp and NANOG-1022bp) extension, after an initial denature step of 94 °C for 5 min. Final extension at 72 °C for 10 min was performed. The PCR products were separated on 1.5% agarose gel electrophoresis. GAPDH was used as an internal control. The sequences of primers (synthesized by Sangon Biotech (Shanghai) Co., Ltd.) were presented in Table 1.

**Table 1:** Primers for TFs (*OCT4*, *SOX2*, *NANOG*) and ecto-, endo-, and mesoderm genes by RT-PCR

Gene	Primer sequence (5′-3′)	Products (bp)
*GAPDH*	For: GAAGGTGAAGGTCGGAGTC	226
	Rev: GAAGATGGTGATGGGATTTC	
*OCT4-I*	For: CGGGCTGATGGGCAAGTT	247
	Rev: GGGCAGGAAGGATGGGTAA	
*OCT4-II*	For: CGCAAGCCCTCATTTCAC	1143
	Rev: GGCAGGCACCTCAGTTTG	
*NANOG*	For: TTTTCCAGTCCACCTCTT	1022
	Rev: GAAGGATTCAGCCAGTGT	
*SPARC*	For: CCTGATGAGACAGAGGTGGTGG	349
	Rev: GCTTGTGGCCCTTCTTGGTG	
*TBX20*	For: AAGGAGGCGACGGAGAACA	289
	Rev: TCCTGCCCGACTTGGTGAT	
*TUBB3*	For: AGCTCAACGCTGACCTGC	499
	Rev: GCCTCGGTGAACTCCATCT	
*WNT1*	For: CCGATGGTGGGGTATTGTG	500
	Rev: TCATGAGGAAGCGCAGGTC	
*Brachyury*	For: AAGAACGGCAGGAGGATG	373
	Rev: TCTCAGGGAAGCAGTGGC	
*Sox2*	For: ATGGACAGTTACGCGCACAT	268
	Rev: GACTTGACCACCGAACCCAT	

### BM-MSC phenotype characterization by flow cytometric analysis

The MSC phenotype with typical surface markers as previously described was evaluated by flow cytometry (FCM) on a BD FACS Calibur flow cytometer. Fluorescein isothiocyanate (FITC)-conjugated anti-human CD29 (CD29 FITC), CD90 FITC, and phycoerythrin (PE) anti-human CD105 (CD105 PE), CD166 PE were obtained from eBioscience and Biolegend, respectively. The hematological lineage-specific markers such as anti-human CD34PerCP, CD45FITC, HLA-DRPerCP, CD14 FITC, and corresponding isotype controls were purchased from Becton Dickinson (San Jose, CA, USA). The optimal generation (P4) MSCs were resuspended in PBS to prepare a cell suspension of 1 × 10^6^/ml. The expression levels of surface markers in other passage MSCs were also tested by the antibody above. Cell Quest software (Becton Dickinson, USA) was used for data acquisition and analysis. At least 5000 cells were analyzed at a time, and the positive rate of surface markers was expressed in percentage (%).

### The identification of BM-MSC stemness features and ESC marker expression

FCM analysis of the P4 BM-MSC preparations was performed with a FACScan instrument (BD Biosciences, Franklin Lakes, NJ, USA) using BD CellQuest Pro software. Cells were stained at 4 °C for 30 min with the following primary antibodies: SSEA-4, TRA-1-60, and TRA-1-81 for detecting ESC surface markers (all of the above antibodies were purchased from Millipore). For the intracellular staining, the cells were fixed, permeabilized, and stained with SOX2-PE (Becton Dickinson), NANOG-PE (R&D Systems), and anti-human OCT3/4 primary antibody, respectively. The cells were washed and then stained with FITC-conjugated goat anti-mouse secondary antibody (GAM-FITC, purchased from Becton Dickinson) and FITC-conjugated goat anti-rat IgG (KPL, USA) at 4 °C for 30 min with light protection. Mouse IgG1-PE and Mouse IgG1-FITC (both were purchased from Becton Dickinson, USA) were served as an isotype control.

### Identification of plasmid transfection and OCT4 overexpression

Transfection of recombinant plasmid pcDNA3.1-OCT4 was carried out using Lipofectamine™ 2000 (Invitrogen) according to the protocol of the manufacturer’s instructions. Before transfection, the plasmid was prepared using PureLink™ HiPure Plasmid DNA Purification Kits (Invitrogen) according to the protocol. The optimal P4 ALL MSCs were used throughout this study. The day before transfection, 2 × 10^5^ BM-MSCs were cultured in Opti-MEM® I (Invitrogen) and transfected with recombinant plasmid twice with pcDNA3.1-OCT4 in 24 h. Then, the cells were passaged at a 1:3 dilution under the same culture condition with DMEM medium in the presence of 500 ng/ml of neomycin (G418 Sulfate, Sangon, Shanghai). The cells with stable OCT4 expression were selected with G418 resistance screening and subcloning. We carried out three times of continuous subcloning to screen out OCT4 overexpression MSCs.

### Cellular immunofluorescence analysis

For cellular immunofluorescence analysis (CIFA), the cells were fixed and permeabilized, then washed with PBS and blocked with 4% of normal goat serum (Boshide, Wuhan, China) for 30 min at room temperature. Then, the cells were incubated with primary antibodies of rat anti-human OCT4 (1:100 dilution, R&D), SSEA-4 (1:100, Millipore), TRA-1-60 (1:100, Millipore), and TRA-1-81 (1:100, Millipore) for 15 min individually. After washing three times, the cells were incubated with the secondary antibodies of FITC-conjugated goat anti-mouse IgG and FITC-conjugated goat anti-mouse IgM (KPL, 1:200 dilution). 4′6-Diamidino-2-phenyl indole (DAPI, Kaiji, China) was used for nuclear staining. Fluorescence staining was observed under a fluorescence microscope and photographed.

### Data analysis

The data presented in this study were statistically analyzed by SAS 9.2 (SAS Institute Inc., Raleigh, NC, USA), and the figures were processed by Microsoft Excel. The data including the expression levels of surface markers and stemness-related TFs (OCT4, SOX2, NANOG) were described as mean ± standard deviation (SD). Analysis of variance was performed for the comparison of the parameters between the two groups at different passages. *P* value < 0.05 was considered to be statistically significant.

## Results

### Characterization of BM-MSCs with flow cytometry and growth evaluation

**Fig. 1:**
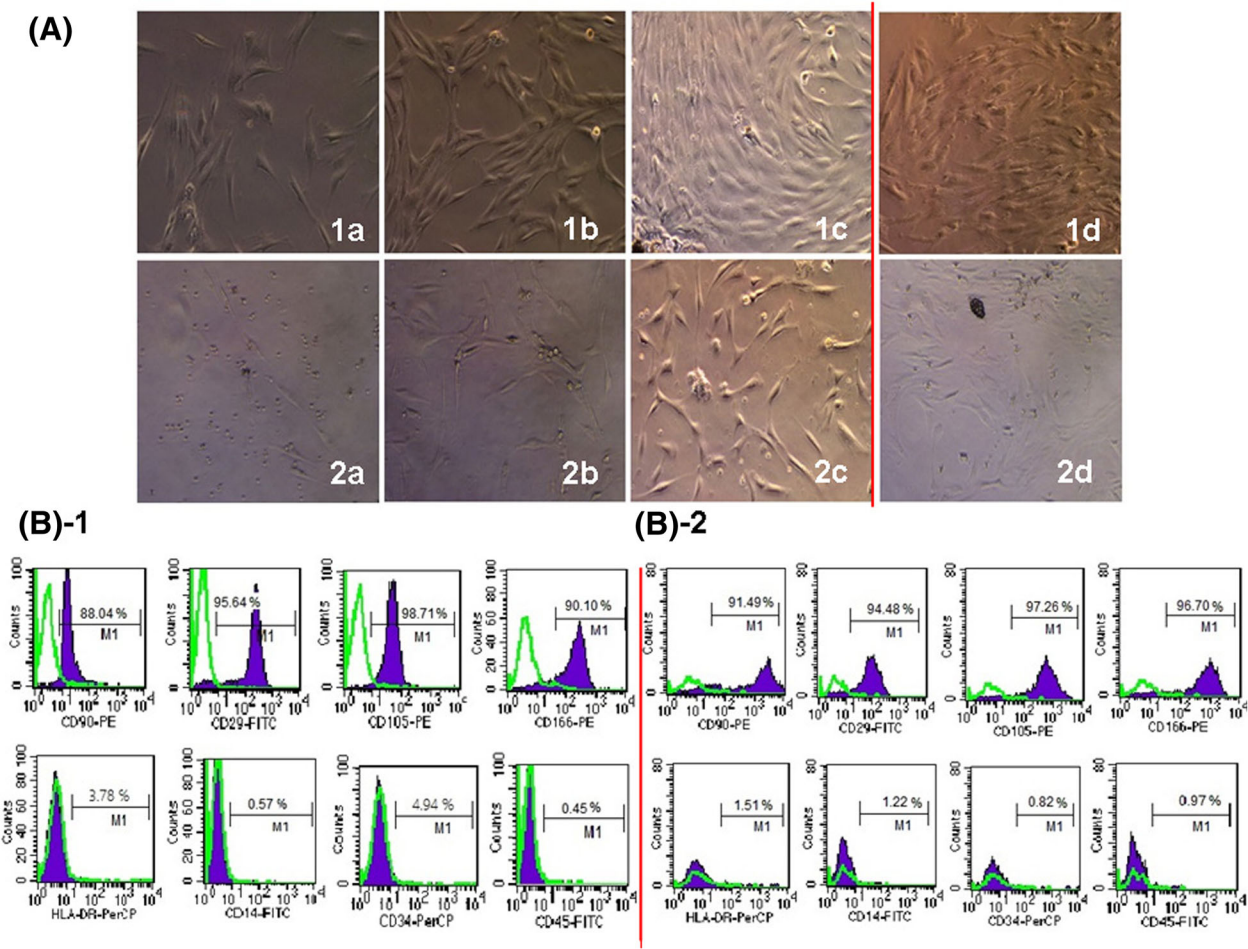
Characterization of cell surface markers and morphology of BM-MSCs. **A** The morphology of ALL MSCs (1a–1d) and AA MSCs (2a–2d). The morphology of the primary passage (P0) and the P4 passage MSCs of ALL (upper panel) and AA (lower panel) at different time points (primary culture—a: day 7, b: day 10, c: day 14; passage culture—d: day 7 P4 MSCs) (× 10). **B-1**, **B-2** Cell surface markers of P4 MSCs were analyzed using FCM. Antibodies against CD90, CD29, CD105, and CD166 (upper panel), and CD34, CD45, CD19, and HLA-DR (lower panel) were used to characterize ALL MSCs (**B-1**) and AA MSCs (**B-2**). The green curves indicated the isotype controls. The purple curves indicated the expression levels of MSC surface markers

**Fig. 2:**
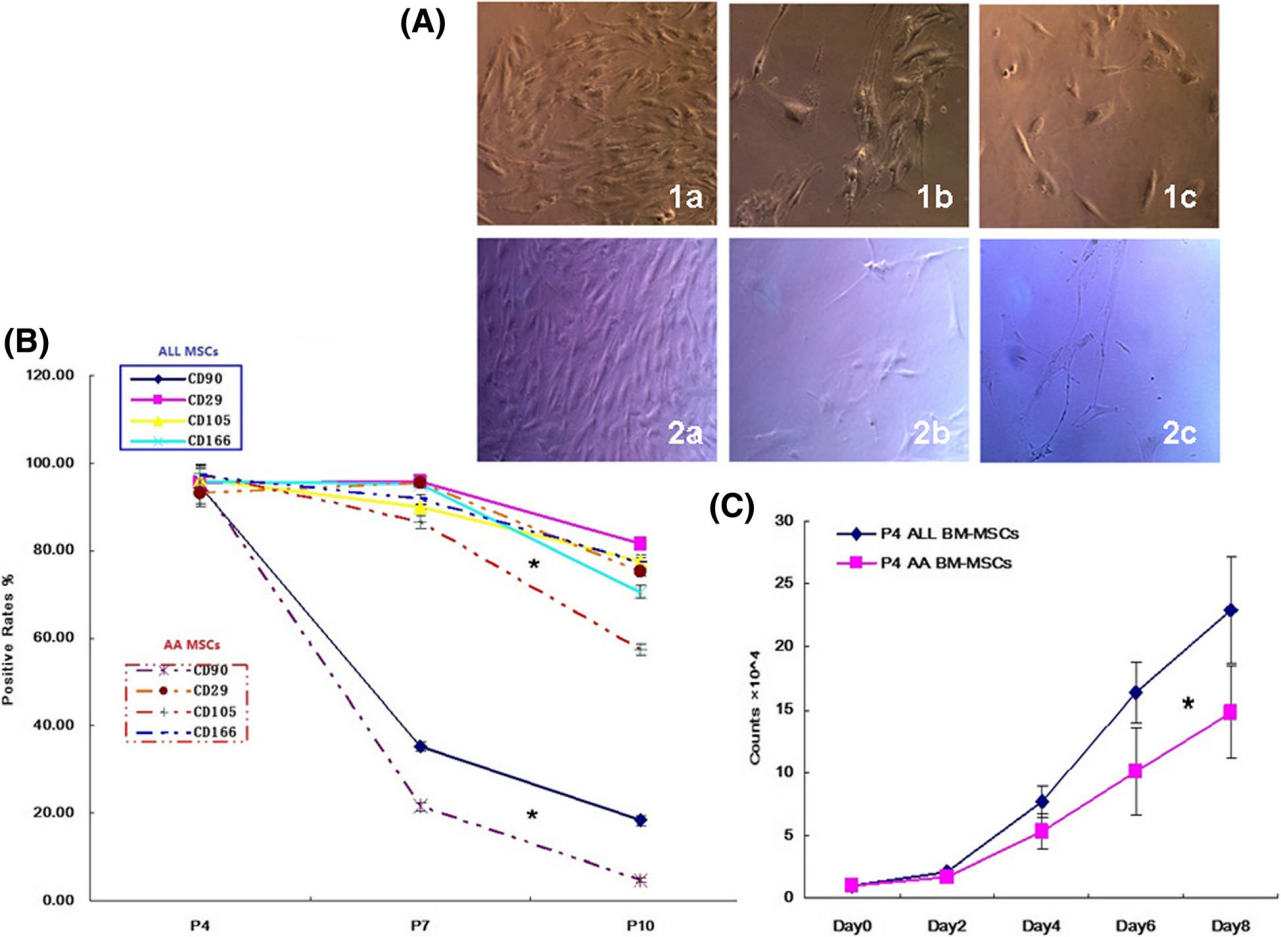
Morphology changes and growth evaluation of the two types of MSCs. **A** Morphology changes of ALL MSCs (1a–1c) and AA MSCs (2a–2c) at P4, P7, and P10 (a: P4, b: P7, c: P10) (× 10). **B** Cell surface markers of P4, P7, and P10 MSCs were analyzed using FCM. Antibodies against CD90, CD29, CD105, and CD166 were used to characterize BM-MSCs (solid dark line for ALL and dotted line for AA MSCs). The results represent mean ± SD (three ALL and three AA MSCs, *P < 0.05). **C** Growth evaluation of P4 MSCs at different time points. The results represented mean ± SD (*P < 0.05)

The morphology changes of MSCs were confirmed by fluorescence microscopy. The results showed that both types of MSCs displayed fibroblast-like, spindle-like, or triangle-like morphology a week later of culture (Fig. 1 (a)). For the primary culture cells, ALL MSCs grew faster than AA MSCs, which reached 80% of confluence in 7–10 days for the former, whereas the latter required more than 2 weeks (Fig. 1(b-c)). However, the passage culture cells grew much faster than their primary ones for both types of BM-MSCs (Fig. 1(d)). There was no significant difference in the morphology between the two types of P0-P4 MSCs (Fig. 1A). Moreover, the surface antigens of BM-MSCs derived from different diseases (i.e., ALL and AA) were similar detected by FCM, showing that both types of MSCs were positive for CD90, CD29, CD105, and CD166 and negative for HLA-DR, CD34, CD45, and CD19, respectively (Fig. 1B-1, B-2), which indicated that both types of MSCs expressed typical mesenchymal surface markers. The expression levels of CD90, CD29, CD105, and CD166 for the optimal P4 ALL MSCs were 94.85 ± 4.81%, 95.53 ± 2.07%, 96.27 ± 2.94%, and 95.77 ± 1.90%, respectively, which were comparable to those derived from AA patients showing 95.85 ± 1.18%, 93.06 ± 2.37%, 97.71 ± 0.92%, and 97.17 ± 2.05%, respectively, for each of the markers described above. The expression levels of CD34, CD45, CD14, and HLA-DR for the former were 0.95 ± 0.28%, 0.83 ± 0.26%, 1.02 ± 0.47%, and 0.92 ± 0.25%, respectively, which were also similar to those derived from the latter showing 0.89 ± 0.09%, 1.09 ± 0.81%, 1.10 ± 0.20%, and 1.05 ± 0.43%, respectively, for individual hematopoietic cell markers (Fig. 1B). However, the proliferative capacity of cells from P4 AA MSCs was much slower than that from P4 ALL MSCs (*P* < 0.05) (Fig. 2c). Moreover, the expression level of specific surface markers such as CD90, CD29, CD105, and CD166 decreased with passaging (Fig. 2b), especially the surface marker CD90, with the expression reduced from 94.85 ± 4.81% (ALL MSCs) and 95.85 ± 1.18% (AA MSCs) at P4 to 18.33 ± 1.13% and 4.69 ± 0.48% at P10 (*P* < 0.05) (Fig. 2b). The above results indicated that ALL MSCs had a better proliferative potential and were more suitable for the subsequent transfection experiment.

### Effect of OCT4 overexpression on cell morphology and proliferation of MSCs

For the primary culture cells, ALL MSCs grew much faster than AA MSCs, with 10 days of PDT for the former, vs. 16 days of PDT for the latter. However, the passage culture cells grew much faster than their primary culture ones for both types of BM-MSCs. AA MSCs began to lose their typical morphology and became flat at P7, whereas those cells from ALL retained the ability to continue to expand until P10 then became flat (Fig. 2a). The expression levels of specific surface markers were decreased with passaging for each type of cells (Fig. 2b). However, OCT4-overexpressing MSCs still retained the morphology of MSCs after long-term culture (Fig. 4d) whereas those lacking expression of OCT4 gradually lost the typical morphology, which indicated that ectopic expression of OCT4 not only promoted the proliferative rate of MSCs, but also maintained the morphology of them.

### Stable expression of OCT4 in MSCs by recombinant plasmid transfection

**Fig. 3:**
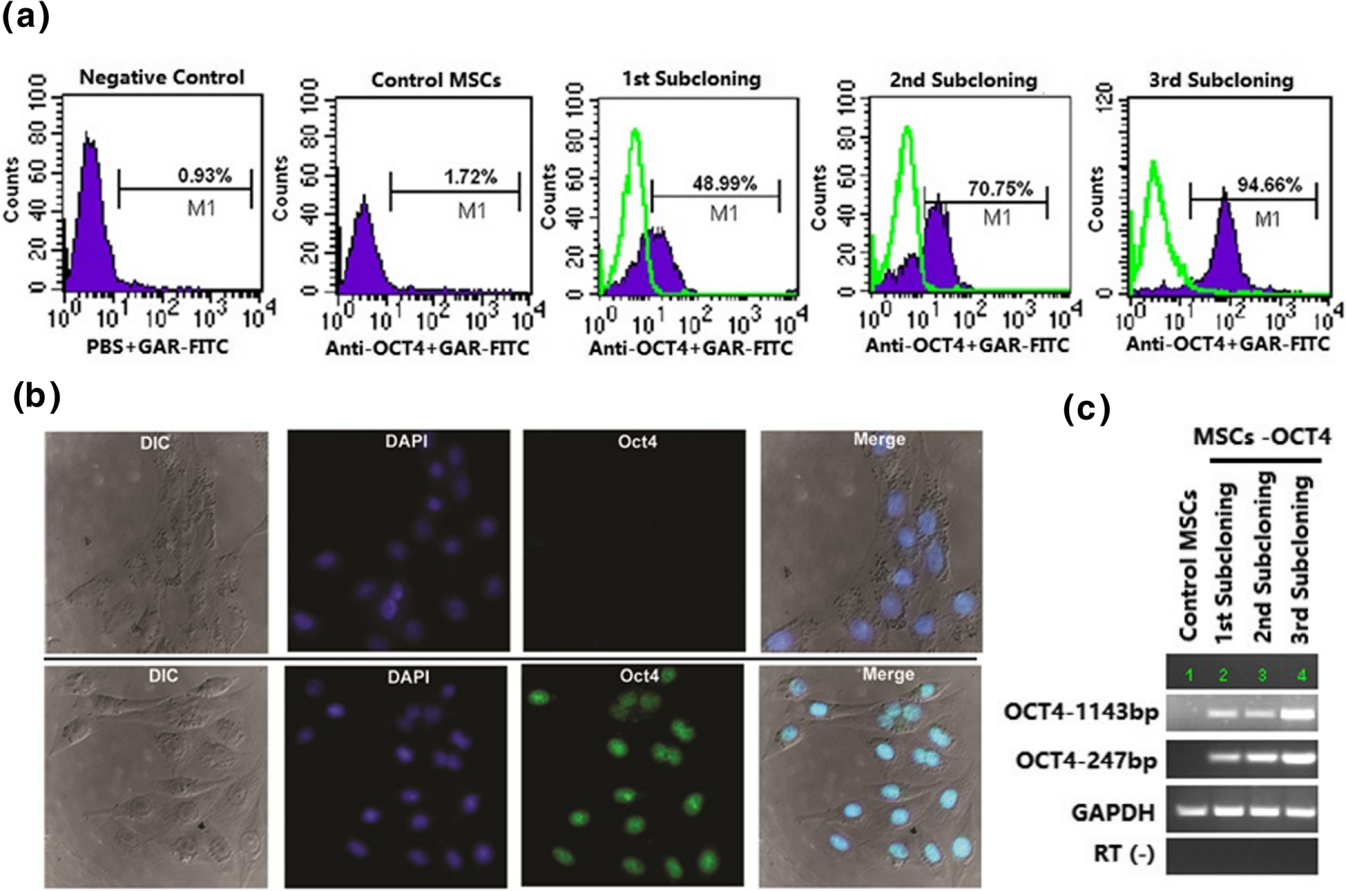
Identification of OCT4 expression in ALL MSCs. **a** The expression of OCT4 protein was analyzed with FCM at various subcloning time. The green curves indicated the isotype antibody control. Control MSCs refer to the untransfected parental ALL MSCs. Negative control refers to the antibody replaced by PBS. **b** The expression of OCT4 was determined by CIFA in transfected MSCs (the upper panel refers to the untransfected parental ALL MSCs, while the lower panel refers to stably transfected MSCs) (the nuclei are stained with DAPI). **c**. OCT4 mRNA was analyzed by RT-PCR at various subcloning times to verify the FCM data. OCT4-1143bp indicates the full length of OCT4 ORF sequence, and OCT4-247bp represents the specific length of OCT4. GAPDH served as an internal control. RT(-) refers to the template replaced by water

The recombinant plasmid was successfully transfected into ALL BM-MSCs with Lipofectamine 2000 under the co-culture pressure of G418, which was confirmed by CIFA analysis (Fig. 3b). Moreover, the expression level of recombinant protein OCT4 gradually increased with subcloning times, with the expression levels of 49.99% in the first subcloning, 70.75% in the second subcloning, and 94.66% in the third, whereas it was expressed only 1.72% in the untransfected parental BM-MSCs (Fig. 3a). The expression of OCT4 mRNA was also detected by RT-PCR. The results showed that the significant elevation of OCT4 mRNA in OCT4 overexpression MSCs was observed with the expression of both the full-length fragment (1143bp) and the specific fragment (247bp) as compared to that of the control MSCs (Fig. 3c), indicating that stable expression of OCT4 could be efficiently mediated by plasmid transfection.

### Effect of OCT4 overexpression on the expression of stemness-related transcription factors

**Fig. 4:**
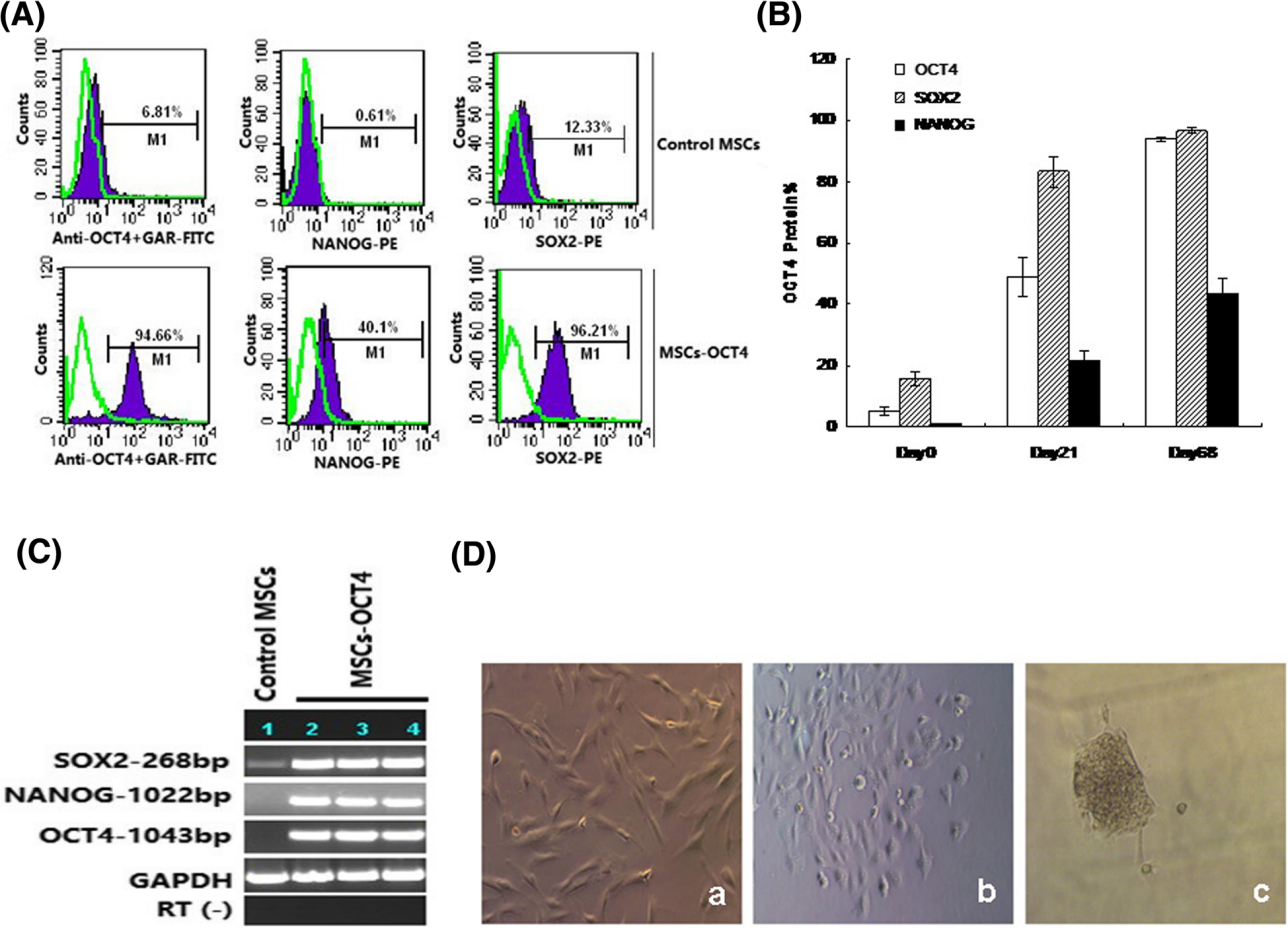
Effect of OCT4 overexpression on the expression of stemness-related transcription factors (TFs) by FCM and RT-PCR. **A** Comparison of the expression levels of stemness-related TFs (NANOG, SOX2, OCT4) by FCM between the OCT4 overexpression and the parental untransfected MSCs. **B** The expression levels of stemness-related TFs detected by FCM at different subcloning time points in OCT4 overexpression and untransfected MSCs. The results represented mean ± SD. **C** mRNA of the three TFs were analyzed by RT-PCR to verify the FCM data. GAPDH serves as an internal control. RT(-) refers to the template replaced by water. Lanes 2–4 refer to the three different ALL samples with OCT4 stable overexpression. **D** The morphology changes of ALL MSCs after transfection with OCT4 (b) and after the third time subcloning (c), and (a) refers to the untransfected P4 MSCs (× 10)

Compared with the untransfected parental MSCs, those with stable expression of OCT4 expressed the embryonic stem cell (ESC) characteristic transcripts such as NANOG and SOX2, from 0.63 ± 0.37% and 14.34 ± 2.44% to 39.39 ± 1.85% and 91.45 ± 4.56%, respectively (Fig. 4a). The expression of the above three TFs (NANOG, OCT4, and SOX2) were increased with the subcloning times, showing that the expression increased from 21.27 ± 3.55%, 48.90 ± 6.46%, and 83.33 ± 4.89% on day 21 to 43.82 ± 4.58%, 93.82 ± 0.85%, and 96.71 ± 0.89% on day 68, respectively, after transfection (Fig. 4b), which indicated that OCT4 expression could upregulate the expression of the other two TFs. The expression of the three TF mRNAs was subsequently confirmed by PCR, which was consistent with the protein expression detected by FCM (Fig. 4c). Interestingly, OCT4 overexpression MSCs cultured in ES medium for more than 1 week showed the typical morphology of ESCs.

### Effect of OCT4 overexpression on the expression of ESC-related surface markers

**Fig. 5:**
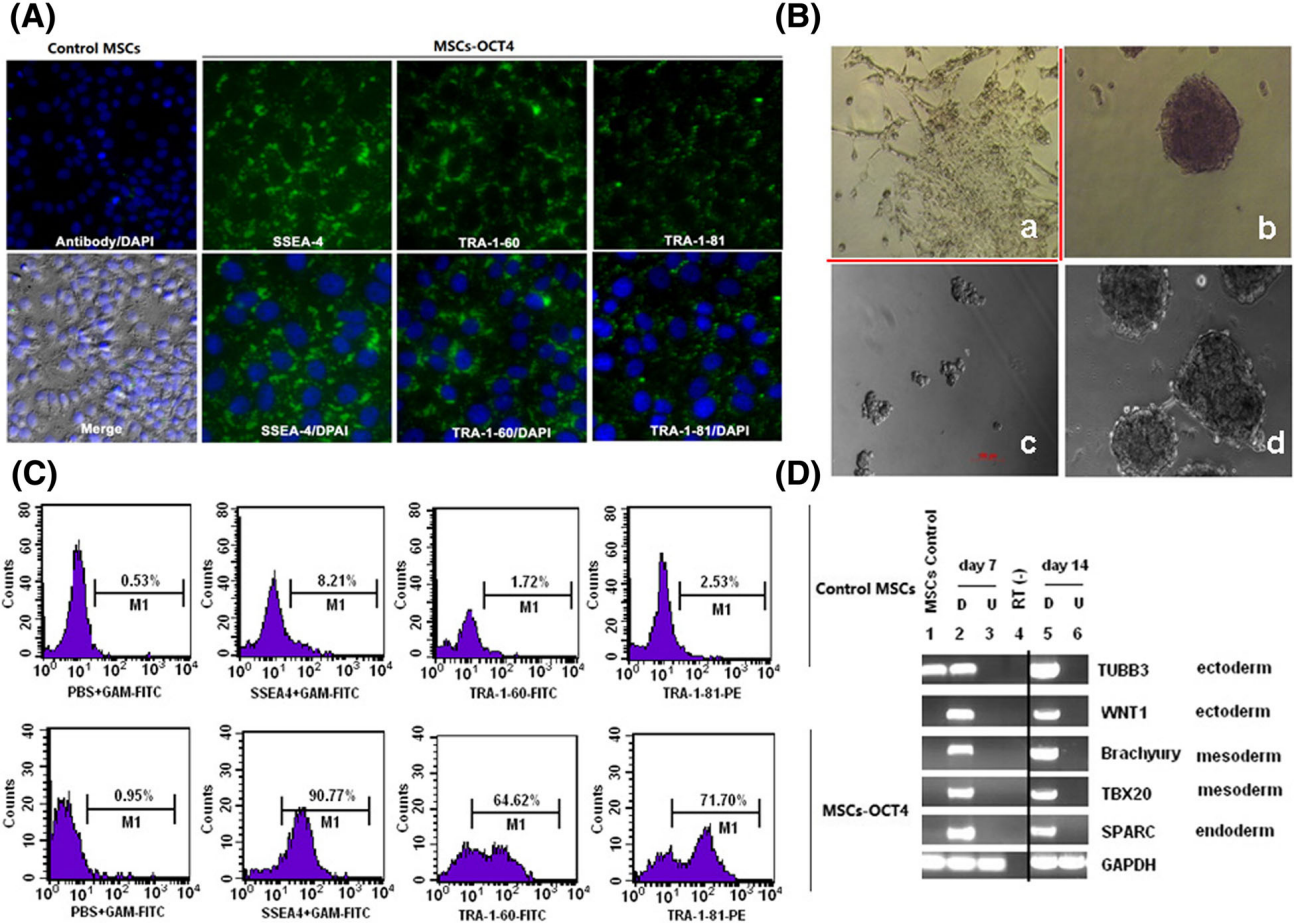
Evaluation the effect of OCT4 overexpression on the expression of ESC-related surface markers and ESC-like properties by FCM, CIFA, and RT-PCR. **A** The expressions of ESC surface markers (SSEA4, TRA-1-60, and TRA-1-81) in OCT4 overexpression ALL MSCs were examined by CIFA. **B** The morphology changes of ALL MSCs with OCT4 stable overexpression in differentiation medium (a: the untransfected MSCs as the control; b: the EBs of MSCs-OCT4; c, d: EBs at different differentiation time points (c: day 3, d: day 7). **C** The expression levels of ESC-related surface markers were verified with FCM in control untransfected (upper panel) and OCT4 overexpression MSCs (lower panel). **D** The expression of ecto-, endo-, and mesoderm genes analyzed by RT-PCR at various differentiation time points (day 7 and day 14) in untransfected and OCT4 overexpression MSCs (“U” refers to undifferentiation and “D” refers to differentiation) (ectoderm gene: TUBB3, WNT1; endoderm gene: SPARC; mesoderm gene: TBX20, Brychury)

In this study, we also examined the expression level of ESC-related surface markers by FCM and CIFA. The CIFA results showed that OCT4 overexpression MSCs expressed ESC characteristic surface markers such as SSEA-4, TRA-1-60, and TRA-1-81 compared to the untransfected parental MSCs (Fig. 5a). The expression levels of SSEA-4, TRA-1-60, and TRA-1-81 in OCT4 overexpression MSCs were increased from 3.33 ± 2.35%, 1.59 ± 1.29%, and 1.46 ± 0.86% to 84.98 ± 9.2%, 57.28 ± 6.72%, and 75.88 ± 7.35%, respectively (Fig. 5c). In addition, the above MSCs showed typical morphology of ESCs (Fig. 5b), had the potential to form embryonic bodies (EBs) ex vivo, and expressed ectoderm (TUBB3, WNT1), mesoderm (Brachyury, TBX20), and endoderm (SPARC) genes (Fig. 5d). The above results indicated that the ectopic OCT4 overexpression MSCs acquired ESC-like properties.

## Discussion

Mesenchymal stromal cells (MSCs), a group of cells with high plasticity, can be isolated from various sources, especially from the bone marrow, which is one of the most common tissues used for isolation [1, 2]. MSCs have the following three characteristics: express a specific set of cell surface markers, exhibit plastic adherence, and have the ability to differentiate in vitro [11]. Owing to their potential for multi-lineage differentiation and immunomodulatory properties [2, 3], MSCs are attractive candidates for cell therapy in immune disorders treatment and the field of regenerative medicine.

Although BM-MSCs are relatively easy to obtain, they are only available in limited amounts, accounting for around 0.01% of all nucleated bone marrow cells. Therefore, cell expansion in vitro is necessary for its potential clinical applications. However, due to the limited ex vivo proliferative capability of BM-MSCs, stemness properties and differentiation potential were gradually lost during expansion [6, 12]. In addition, disease-specific or tissue-specific MSCs may have different biological characteristics such as surface markers, immunophenotype, and proliferation and differentiation potentials [5, 6]. In this study, we had compared the growth properties and surface marker expression between the two types of MSCs. We found that the AA MSCs had a lower proliferative capacity than that of ALL MSCs and needed longer time for expansion; meanwhile, the specific surface markers especially CD90 were decreased with passaging, which were consistent with the results reported in the previous literature [13, 14]. Moreover, these two types of BM-MSCs mildly expressing the stemness marker SOX2 and extremely low expression of SSEA4 if any lacked the expression of ESC characteristic markers such as OCT4, NANOG, and the hematopoietic antigens such as CD34 and CD45.

OCT4 is one of the important transcription factors required to maintain the pluripotency and self-renewal of ESCs. OCT4-expressing MSCs displayed high proliferative capacity whereas those with OCT4 knockdown presented significantly low growth rates [15]. Progenitor cells increased proliferation after ectopic expression of OCT4 [2]. We have investigated the effect of high expression OCT4 on the stemness characteristics of ALL MSCs, and it was clearly demonstrated that OCT4 overexpression improved proliferative capacity, which was similar to the results reported in the previous literatures [2, 15] Meanwhile, the OCT4 overexpression cells presented ESC-like characteristics expressing higher levels of stemness-related markers such as NANOG, SOX2, SSEA-4, and TRA-1-60. Moreover, these cells have the ESC characteristic features with ESC-like morphology and high expression of specific surface markers and have the potential to form EBs and expressed triembryonic genes ex vivo, suggesting that OCT4 may play an important role in maintaining stemness of MSCs and the ectopic OCT4 overexpression MSCs acquired ESC-like properties. However, whether or not the above OCT4 overexpression cells could be engineered as potential induced pluripotent stem (iPS) cells requires further study.

OCT4 belongs to a member of the POU transcription factor family. It has multiple transcription initiation sites, which can activate or inhibit gene expression or synergize with different protein factors to activate or inhibit the expression of upstream and downstream genes [16]. It has been reported that at least 353 target genes are co-owned by OCT4, SOX2, and NANOG, and the promoter binding sites of these three transcription factors are close to each other, suggesting that these 3 transcription factors synergistically regulate the expression of their target genes [16]. Our findings have shown that OCT4 can upregulate the expression of NANOG and SOX2, improve the proliferative capacity of MSCs, and maintain the morphology of them, which indicates that OCT4 might be an important drive gene in the regulating process and the stemness properties of MSCs.

Recently, it has been shown that the introduction of the defined factors into human somatic cells may result in the generation of induced pluripotent stem (iPS) cells similar to hESCs in morphology, proliferation, surface antigens, gene expression, epigenetic status of pluripotent cell-specific genes, and telomerase activity [17]. It is also reported that single OCT4 can generate iPS cells [8, 18]. Transduction using virus vectors such as retrovirus and lentivirus is the most popular method in iPSC reprogramming albeit with the risks of foreign gene integration and oncogene activation. In addition, it has been demonstrated that it is difficult to transfect with Lipofectamine™ 2000 due to low transfection efficiency [19]. Thus, we performed twice consecutive transfections combining with limited dilution culture with G418 (500 ng/ml) to improve the transfection efficiency and to increase the expression of OCT4. Our study showed that stable expression of OCT4 could be efficiently mediated by plasmid transfection; moreover, single OCT4 overexpression in human MSCs not only promoted cell proliferation, but also maintained the morphology of MSCs, suggesting that OCT4 overexpression maintained the stemness of BM-MSCs. This study also demonstrated a possible approach to use stemness-related transcription factors to engineer the high proliferation and quality MSCs for clinical applications.

Although BM-MSCs have great advantages, their clinical applications are limited by many factors. One of the major challenges is to obtain an adequate number of cells as these cells were found to get senescence during in vitro expansion or at higher passages. One of the reasons for this problem is the decrease of telomerase activity [20]. It also has been reported that BM-MSCs become senescent during subculturing or long-term culture, accompanied with proliferative capacity decrease and morphological alterations [21]. Our results have shown that MSCs especially MSCs derived from aplastic anemia progressively have decreased the expression of CD90 and CD29 and lost the typical morphology at higher passages, which is to some extent consistent with what has been reported in the literature [14, 22]. Furthermore, the microenvironment of AA was in disorder which may damage the quality of MSCs and make them easy to become senescent. In our study, we found that ALL MSCs got senescence at higher passages as well, whereas OCT4 overexpression MSCs could maintain the morphology and proliferation capacity for long-term culture. However, the underlying mechanism on the senescence of MSCs needs to be further studied to better understand its biological function.

The shortcomings of this study include the following: firstly, the underlying molecular mechanisms of OCT4 overexpression promoting cell proliferation and maintaining morphology of MSCs have not been clearly investigated and warranted intensive study; secondly, due to the small sample size of disease-specific BM-MSCs, the differences in biological characteristics and proliferation potential between them need further investigation; finally, whether or not the above ESC-like cells could be engineered as potential iPS cells remains to be answered and is worthy of in-depth study.

## Conclusion

In summary, based on the results we have got, ectopic high expression of transcription factor OCT4 may drive the BM-MSCs into ESC-like cells and affect the “stemness” characteristics of BM-MSCs. OCT4 can upregulate the expression of other stemness-related transcription factors such as NANOG and SOX2.

## Abbreviations

AA: Aplastic anemia patients
ALL: Acute lymphoblastic leukemia
BM-MSCs: Mesenchymal stromal cells derived from bone marrow
CIFA: Cellular immunofluorescence assay
EBs: Embryonic bodies
ESC: Embryonic stem cells
FCM: Flow cytometry
GVHD: Graft-versus-host disease
iPS: Induced pluripotent stem cells
PDT: Population doubling time
TFs: Transcription factors
